# Parastomal hernias successfully repaired using a modified components separation method: two case reports

**DOI:** 10.1186/1752-1947-7-180

**Published:** 2013-07-05

**Authors:** Katsuhito Suwa, Ken Hanyu, Toshiaki Suzuki, Shintaro Nakajima, Tomoyoshi Okamoto, Katsuhiko Yanaga

**Affiliations:** 1 Department of Surgery, Jikei University School of Medicine, Daisan Hospital, 4-11-1 Izumihoncho, Komae-city, Tokyo 201-8601, Japan; 2 Department of Surgery, Jikei University School of Medicine, 3-25-8 Nishishinbashi, Minato-ku, Tokyo 105-0003, Japan

**Keywords:** Parastomal hernia, Components separation method, Incisional hernia, Repair

## Abstract

**Introduction:**

Parastomal hernia is a frequent complication after enterostomy formation. A repair using prosthetic mesh by way of a laparoscopic or open transabdominal approach is usually recommended, however, other procedures may be done if the repair is to be performed in a contaminated environment or when the abdominal cavity of the patient is difficult to enter due to postsurgical dense adhesion. The components separation method, which was introduced for non-transabdominal and non-prosthetic ventral hernia repair, solves such problems.

**Case presentation:**

Case 1. A 79-year-old Japanese woman who underwent total cystectomy with ileal conduit for bladder cancer presented with a parastomal hernia, which was repaired using a keyhole technique. Simultaneously, an incisional hernia in the midline was repaired with a prosthetic mesh. One year after her hernia surgery, a recurrence occurred lateral to the stoma, but it was believed to be difficult to enter the peritoneal cavity because of the wide placement of mesh. Therefore, surgery using the components separation method was performed.

Case 2. A 72-year-old Japanese man underwent an abdominoperineal resection for rectal cancer. At 5 and 12 months after his operation, a perineal hernia and an incisional hernia in the midline were repaired with prosthesis using a transabdominal approach, respectively. Three years after his rectal surgery, a parastomal hernia developed lateral to the stoma. For the same reason as case 1, surgery using the components separation method was performed. No recurrence was observed in either case as of 40 and 8 months after the last repair, respectively.

**Conclusion:**

The components separation method is a novel and effective technique for parastomal hernia repair, especially in cases following abdominal polysurgery or midline incisional hernia repairs using large pieces of mesh. To the best of our knowledge, this is the first report in English on the application of the components separation method for parastomal hernia repair.

## Introduction

Parastomal hernia is the most common stoma complication occurring in 1.8 to 28.3% of end ileostomy and 4.0 to 48.1% of end colostomy [1]. Although classified as an abdominal incisional hernia, the parastomal hernia is quite different from other abdominal hernias because this type of hernia tends to be exposed to contaminated environments, and a part of the hernia orifice is made of the intestinal tract. Also, as midline incisional hernias frequently occur concomitantly with parastomal hernias, some patients already have incisional hernia repairs using large pieces of mesh, which makes it difficult to enter the peritoneal cavity for parastomal hernia repair if necessary. Local fascial repair for parastomal hernias results in recurrence and should be abandoned [2-6] because of tension around the stoma. The components separation method (CSM), which has been suggested as a surgical technique that does not place stress on the atretic portion of the hernia orifice, does not need to enter the peritoneal cavity and can also be performed in contaminated environments. We report two cases of parastomal hernia successfully repaired with a modified CSM following incisional hernia repair with prosthesis.

## Case presentation

### Case 1

**Figure 1:**
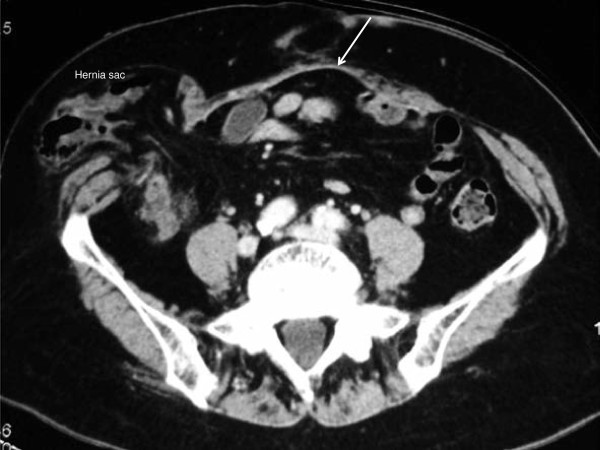
**Preoperative computed tomography image of case 1.** The transverse colon is protruding into the hernia sac lateral to the ileal conduit. A prosthetic mesh (arrow) previously used for a midline incisional hernia is visualized.

A 79-year-old Japanese woman, who had undergone total cystectomy and an ileal conduit diversion for cancer of the urinary bladder, presented with a bulge causing pain on the lateral aspect of the stoma one year after her operation. Computed tomography (CT) revealed a parastomal hernia, for which an operation using a keyhole technique was performed. The operative findings and technique are as follows: To prepare the surgical field, a 24Fr Foley catheter was inserted into the stoma and a balloon was inflated with 10mL of distilled water, and an occlusive dressing with an Ioban™ drape was applied. A midline incision was made to enter the peritoneal cavity, and the transverse colon, which was protruding into the hernia sac lateral to the ileal conduit, was reduced into the peritoneal cavity. The hernia orifice, 3.4×1.7cm in diameter, was observed lateral to the stoma limb, surrounding adhesions were thoroughly dissected, and the hernia repair was performed with a keyhole technique using Composix™ E/X mesh (10.2×15.2cm). For the keyhole, the polypropylene part was trimmed to 1cm wider than the expanded polytetrafluorethylene (ePTFE) part so that only the ePTFE part would be in contact with the ileal conduit to prevent subsequent bowel erosion. Also, simultaneously, an incisional hernia 2.5cm in size was recognized in the midline, which had been repaired with an 11×14cm Composix™ Kugel™ patch. The patient’s postoperative recovery had been good, however, in the first year after her surgery, a hernia recurred on the lateral aspect of the stoma (Figure 1) and an operation was scheduled as she was experiencing pain. Since a large mesh was found just beneath the midline to the right in the peritoneal cavity on CT, entering the peritoneal cavity would be difficult and a modified CSM was planned.

**Figure 2:**
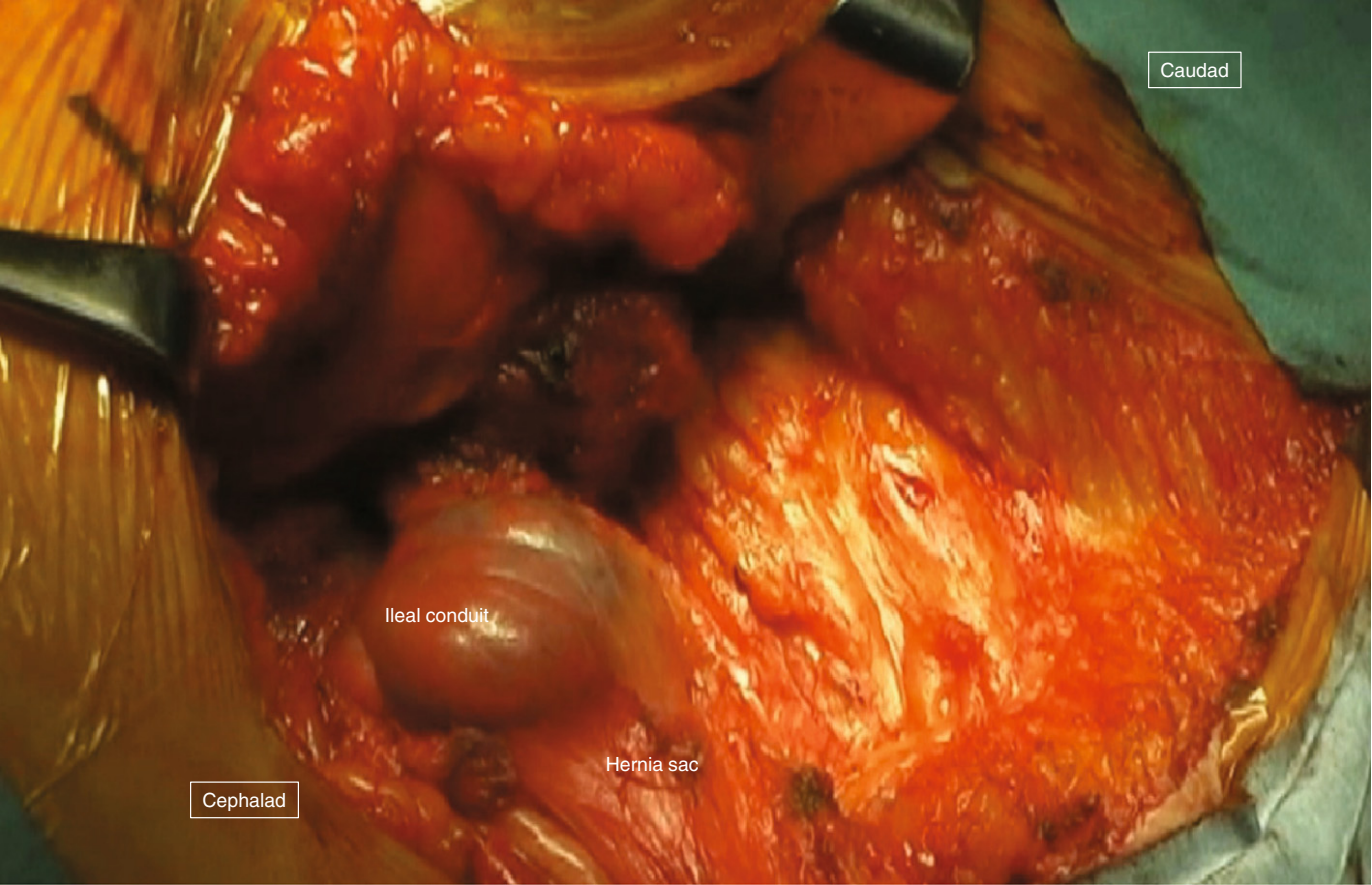
**Operative technique for case 1 (1).** Indigo carmine solution is injected into the ileal conduit, making the boundaries of the hernia sac clear.

**Figure 3:**
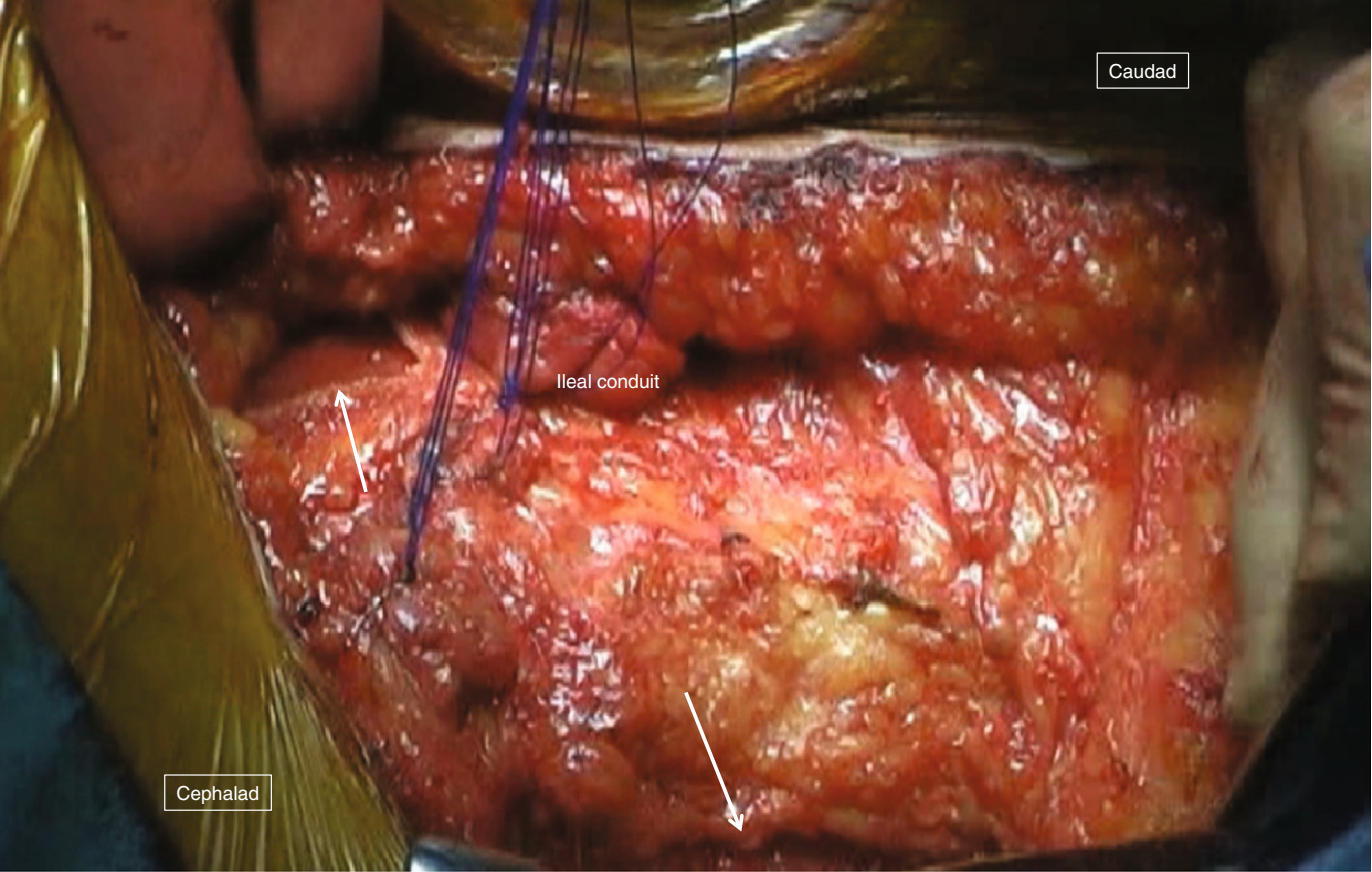
**Operative technique for case 1 (2).** Relaxing incisions (arrow) are made on the anterior sheath of the rectus abdominis muscle and the external oblique aponeurosis, allowing the hernia orifice to be closed without tension.

**Figure 4:**
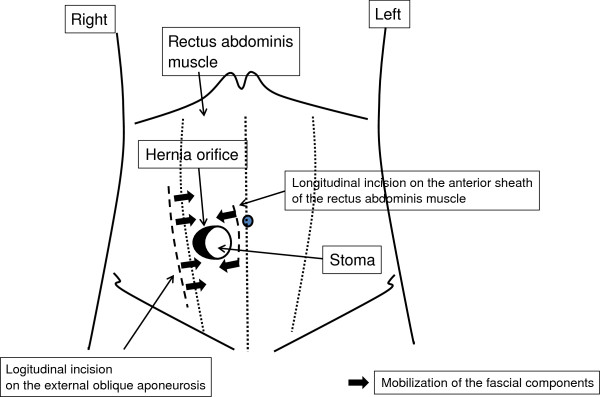
**Schematic explanation of the operative technique for case 1.** Bold arrows indicate the direction of the fascial mobilization.

The surgical field for CSM was prepared in the same fashion as the previous operation. A 12cm semicircular incision was made on the skin 5cm lateral to the stoma. Since the boundary between the subcutaneous hernia sac and the ileal conduit was ill defined, 20mL of indigo carmine solution was injected into the stoma through the preoperatively placed Foley catheter (Figure 2). The hernia sac was dissected intact from the stoma limb and reduced to the preperitoneal space. The hernia orifice was approximately 4cm. A vertically oriented incision parallel with the semilunaris was made 1cm lateral to it on the aponeurosis of the external oblique muscle (8cm) and the anterior sheath of the rectus abdominis muscle also was longitudinally incised for approximately 5cm to allow tension-free closure of the hernia orifice (Figures 3 and 4).

A balloon was inflated inside the stoma, and the fascia around the stoma was tightly closed. The patient recovered well and was discharged on postoperative day 11, and as of 40 months after surgery, she has not experienced any recurrence.

### Case 2

**Figure 5:**
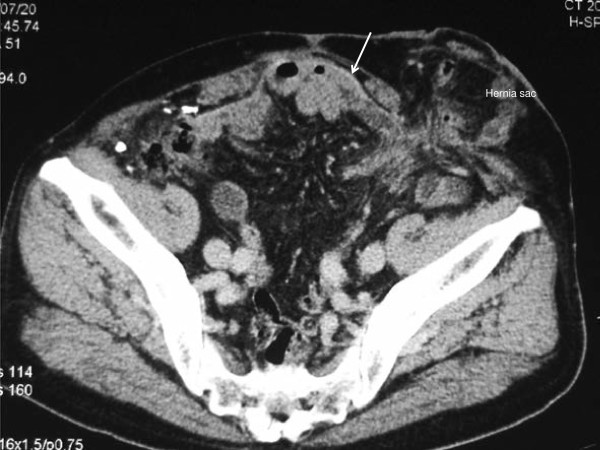
**Preoperative computed tomography image of case 2.** The redundant sigmoid colon is protruding from the stoma's lateral aspect into the hernia sac. A prosthetic mesh (arrow) previously placed for the midline incisional hernia is visualized.

A 72-year-old Japanese man underwent an abdominoperineal resection for rectal cancer. At 5 and 12 months after his operation, a perineal hernia and an incisional hernia in the midline were repaired with prosthesis using a transabdominal approach, respectively. Three years after his rectal surgery, a parastomal hernia developed lateral to the stoma. A CT scan confirmed a hernia sac on the lateral side of the stoma, which was a protruding redundant sigmoid colon (Figure 5). A parastomal hernia was diagnosed and an operation was scheduled, but as with case 1, the patient had previously undergone mesh repair for an incisional hernia as well as abdominal polysurgery, which meant that access to the abdominal cavity was judged as difficult, and a modified CSM was employed.

**Figure 6:**
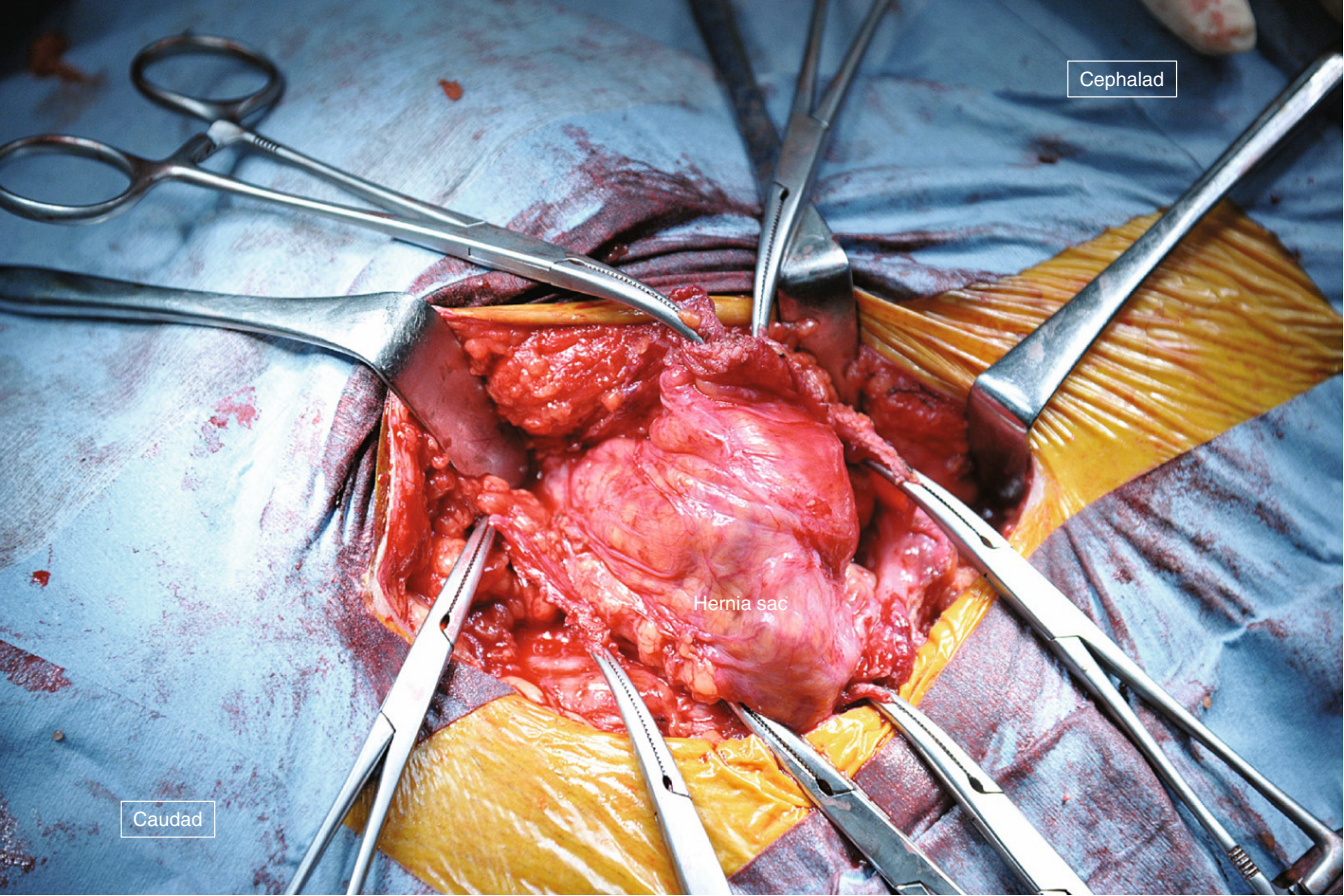
**Operative technique for case 2 (1).** The colon is identified by palpation in continuity with the stoma limb inside the hernia sac on the lateral aspect of the stoma.

**Figure 7:**
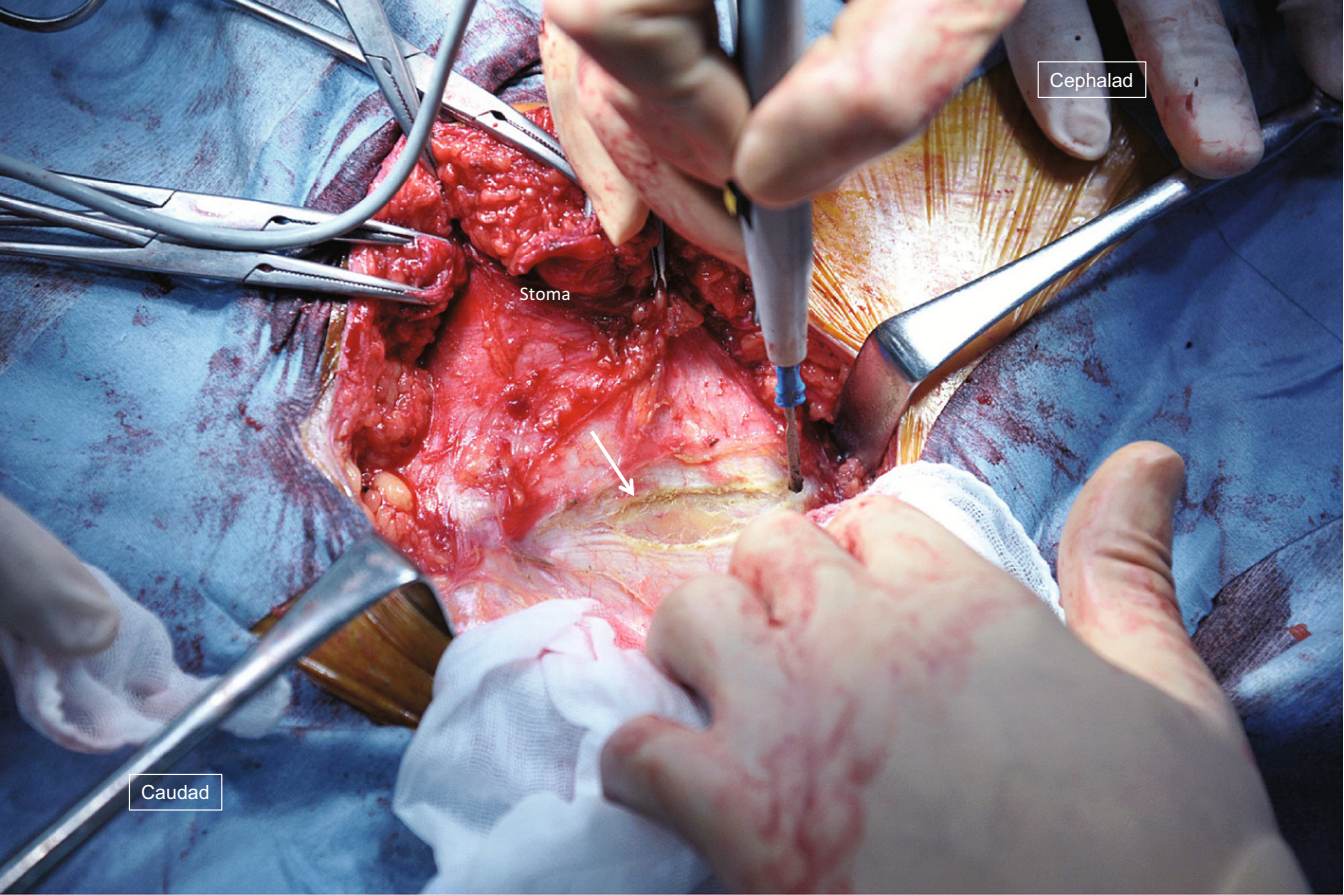
**Operative technique for case 2 (2).** A longitudinal incision (arrow) is made on the external oblique aponeurosis, and the fascia is slid medially.

**Figure 8:**
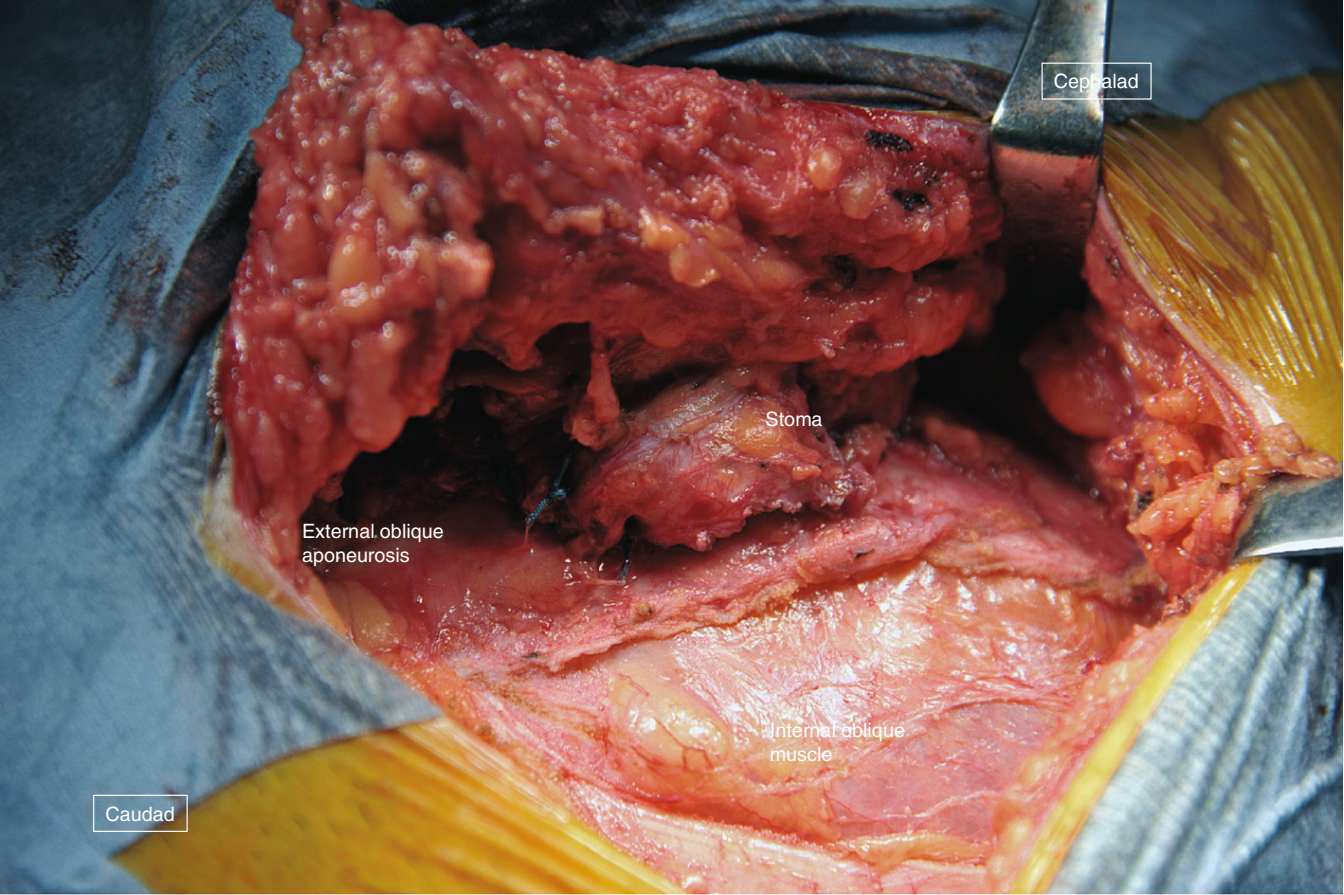
**Operative technique for case 2 (3).** The hernia orifice is closed without tension.

The surgical field was prepared in the same fashion as in case 1, and a semicircular incision was made on the skin 5cm lateral to the stoma. The hernia sac was located on the stoma’s lateral aspect, and palpation confirmed that the inside was adhered to the intestine (Figure 6). The hernia sac was opened to enter the peritoneal cavity, where the sigmoid colon continuing from the stoma limb was found to be adhered to the hernia sac. The adhesion was dissected, the sigmoid colon was reduced to the peritoneal cavity, and the hernia sac was closed. Then, in order to close the fascial defect, a 10cm incision was made longitudinally on the aponeurosis of the external abdominal oblique muscle (Figure 7), and a repair was done without tension using the modified CSM (Figure 8). The fascia closure was the same as for case 1: a balloon was inserted into the stoma, and the fascia was tightly closed around the stoma. In this case, it was clear that the patient’s abdominal wall was weakened due to abdominal polysurgery, and a lightweight polyester mesh was used in the onlay method to provide further reinforcement. His postoperative recovery was uneventful and the patient was discharged on postoperative day 9, and as of 8 months after the repair, he remains well and without any recurrence.

## Discussion

Parastomal hernia is an incisional hernia related to an abdominal wall stoma [7] and is the most common complication of the stoma which occurs in 1.8% to 28.3% of end ileostomies, up to 6.2% of loop ileostomies, 4.0 to 48.1% of end colostomies, and up to 30.8% of loop colostomies [1]. Many parastomal hernias are asymptomatic, but may produce problems ranging from mild parastomal discomfort to life-threatening complications such as strangulation, perforation and obstruction. The contents of the hernia may be omentum, small intestine, stomach and colon. Many patients suffer from parastomal pain, intermittent obstructive episodes and difficulty with appliance application that may result in skin irritation. Repair methods include localized fascial suturing, relocation, and use of prosthetic meshes, but the former two are not recommended as they have a high likelihood of recurrence. The literature shows that direct fascial sutures represented by the Thorlakson technique [8] have a recurrence rate of 46 to 100% [1]. In the report of Allen-Mersh and Thomson [9], the recurrence rate of relocation with creation in the abdominal wall was as high as 57% on the same side of the stoma, which increases to 86% if created on the opposite side. Hansson *et al.*[10] conducted a systematic review of the literature on parastomal hernia and stated that simple fascial suturing should not be used, and moreover that the Sugarbaker technique should be employed in laparoscopic repair [11]. However, in a contaminated environment, or for patients who have already had prosthesis for incisional hernias making it difficult to enter the abdominal cavity, it is necessary to consider repair other than using an open transabdominal approach or with prosthetic meshes. In the cases described here, we decided not to operate using a transabdominal approach or to use prosthetic meshes, but to use a modified CSM as a tension-free fascial suture technique.

The CSM is a technique for an abdominal wall hernia repair reported by Ramirez *et al.*[12] in 1990. His anatomic studies revealed that separating the external oblique fascia with an incision just lateral to the linea semilunar allows creation of a plane between the external oblique and internal oblique muscles all the way to the posterior axillary line if necessary. This method produces immediate mobility of the ipsilateral rectus abdominis muscle-internal oblique-transversus abdominis muscle complex and allows significant freedom for medial transposition of this entire complex. Surgical dissection and separation in this avascular plane totally preserves the innervation of the rectus abdominis muscles, because the intercostal nerves supplying this muscle run deep to the fascia of the internal oblique muscle, which is lateral to the linea semilunaris. This innervated muscle complex can be mobilized approximately 4cm at the subxiphoid level, approximately 8cm at the waist region, and 3cm in the suprapubic region on each side, allowing the surgeon to reconstruct defects up to 16cm in width at the waist level. An additional small amount of medial advancement (2cm on each side) can be obtained by separating the deep surface of the rectus abdominis muscle from the underlying posterior rectus sheath above the arcuate line. This procedure can contribute an additional 2cm of medial advancement for each muscle complex. Therefore, it is possible to close extremely large midline defects in a single operation.

In our cases, longitudinal incisions were made on the aponeurosis of the external oblique muscle lateral to the stoma and the anterior sheath of the abdominal rectus muscle medial to the stoma, which allowed tension-free closure of the hernia orifice enveloping the stoma edge. To the best of our knowledge, there is no report of the CSM being applied to repair a parastomal hernia besides our report in Japanese [13]. Unlike the Thorlakson technique, which is considered not suitable, this technique enables tension-free closure of the hernia orifice, and thus seems applicable to parastomal hernias. The technical challenge of this procedure includes the degree of tightness of the fascia for closure of the hernia orifice, and the suturing of the fascia to the intestine may be controversial. However, the use of balloon inflation inside the stoma for the closure of the fascia seems to abrogate intestinal stenosis or recurrence.

## Conclusion

The modified components separation method is an effective technique for the repair of parastomal hernia in patients who have undergone surgery using prosthetic repair for incisional hernias or abdominal polysurgery.

## Consent

Written informed consent was obtained from the patients for publication of this manuscript and any accompanying images. A copy of the written consent is available for review by the Editor-in-Chief of this journal.

## Competing interests

The authors declare that they have no competing interests.

## Authors’ contributions

KS was involved in the conception of the report, literature review, manuscript preparation, editing and submission. KH, TS, SN, TO and KY were responsible for the manuscript critique and review. All authors have read and approved the final manuscript.
